# Novel coronavirus-like particles targeting cells lining the respiratory tract

**DOI:** 10.1371/journal.pone.0203489

**Published:** 2018-09-05

**Authors:** Antonina Naskalska, Agnieszka Dabrowska, Paulina Nowak, Artur Szczepanski, Krzysztof Jasik, Aleksandra Milewska, Marek Ochman, Slawomir Zeglen, Zenon Rajfur, Krzysztof Pyrc

**Affiliations:** 1 Malopolska Centre of Biotechnology, Jagiellonian University, Krakow, Poland; 2 Microbiology Department, Faculty of Biochemistry, Biophysics and Biotechnology, Jagiellonian University, Krakow, Poland; 3 Department of Skin Structural Studies, Medical University of Silesia in Katowice, School of Pharmacy with the Division of Laboratory Medicine, Sosnowiec, Poland; 4 Department of Cardiac Surgery and Transplantology, Silesian Center for Heart Diseases, Zabrze, Poland; 5 Institute of Physics, Faculty of Physics, Astronomy and Applied Computer Sciences, Jagiellonian University, Krakow, Poland; Deutsches Primatenzentrum GmbH - Leibniz-Institut fur Primatenforschung, GERMANY

## Abstract

Virus like particles (VLPs) produced by the expression of viral structural proteins can serve as versatile nanovectors or potential vaccine candidates. In this study we describe for the first time the generation of HCoV-NL63 VLPs using baculovirus system. Major structural proteins of HCoV-NL63 have been expressed in tagged or native form, and their assembly to form VLPs was evaluated. Additionally, a novel procedure for chromatography purification of HCoV-NL63 VLPs was developed. Interestingly, we show that these nanoparticles may deliver cargo and selectively transduce cells expressing the ACE2 protein such as ciliated cells of the respiratory tract. Production of a specific delivery vector is a major challenge for research concerning targeting molecules. The obtained results show that HCoV-NL63 VLPs may be efficiently produced, purified, modified and serve as a delivery platform. This study constitutes an important basis for further development of a promising viral vector displaying narrow tissue tropism.

## Introduction

Virus like particles (VLPs) have recently emerged as promising and versatile molecular biology tools. Formed by structural viral proteins which have an inherent property for self-assembly, these structures not only mimic the morphology of the native virus but can also transduce permissive cells. Devoid of viral genetic material, VLPs do not replicate within the host cell, but can be used as carriers for nucleic acids, proteins or drugs. Numerous studies have demonstrated that VLPs originating from different viral species may be produced using eukaryotic expression systems (reviewed in [1–3]). Coronaviruses—enveloped, positive-stranded RNA viruses that cause common respiratory diseases in humans and a broad variety of diseases in animals are also able to form such structures. Successful production of coronavirus-like particles has been reported for severe acute respiratory syndrome coronavirus (SARS-CoV) [4–8], mouse hepatitis virus (MHV) [9–13], avian infectious bronchitis virus (IBV) [14], porcine transmissible gastroenteritis virus (TGEV) [15, 16] and porcine epidemic diarrhea virus (PEDV) [17].

The majority of studies describing the production of coronaviral like particles focus on two main threads—particle assembly and immunogenicity assessment. The propitious property of being tailorable and non-infectious renders VLPs a handy tool for studying not only requirements for efficient trafficking, assembly and release of viral particles, but also interactions with cellular receptors. Numerous studies using coronaviral like particles provided valuable data on structure of SARS [6, 8, 18], MHV [9, 10, 12, 13, 19], IBV [14] and TGEV [16]. The second branch of VLP investigation focuses on their use as vaccine candidates. Due to the repetitive exposition of surface antigens and their particulate structure, VLPs interact with the immune system similarly to native viruses, inducing humoral and cellular responses. Production of protective antibody titers, as well as induction of cell-mediated immunity induced by VLPs have been shown for animal and human coronaviruses, such as IBV [20, 21], PEDV [17], TGEV [15], SARS [22, 23] and recently for middle east respiratory syndrome coronavirus (MERS-CoV) [24, 25].

In this work we describe the design, production and characterization of VLPs based on structural proteins of human coronavirus NL63 (HCoV-NL63). HCoV-NL63 is a wide-spread virus, causing infections of the lower and upper respiratory tract of varying severity [26]. The HCoV-NL63 virion is composed of membrane (M), envelope (E) and protruding spike (S) proteins, all three shaping the membrane to form the envelope protecting ribonucleocapsid [27]. Recently also ORF3 protein was identified as structural protein of HCoV-NL63, yet its function remains to be elucidated [28]. M is relatively small protein (26 kDa), which spans the viral membrane three or four times. It is essential for virus assembly and budding, as it forms the membrane curvature and interacts with the ribonucleoprotein and other structural proteins (E and S) [29]. E protein (9 kDa) is also engaged in virus assembly and egress [30, 31]. S protein (150 kDa) is anchored in the viral envelope by its C-terminal part, while its large ectodomain trimerize and forms characteristic spikes at the virion’s surface [32]. S protein is responsible for receptor binding and virus entry into host cells [33]. HCoV-NL63 infects exclusively ciliated cells of human respiratory epithelium and for the penetration of the cell it requires interaction between spike protein and angiotensin converting enzyme 2 (ACE2) [34]. This narrow specificity and tropism to respiratory tissue render HCoV-NL63 an interesting basis for development of VLPs, that have not been described yet.

In this manuscript we present the design and production of HCoV-NL63-based VLPs. Several modifications of proteins forming HCoV-NL63 VLP were introduced and their influence on particle assembly and function was tested. Moreover, we have developed a chromatography purification method of HCoV-NL63 VLPs. Finally, we demonstrated that HCoV-NL63 VLPs may serve as highly specific delivery vectors to cells expressing the ACE2 protein.

## Materials and methods

### Cell lines and viruses

Sf9 (*Spodiptera frugiperda*, ATCC: CRL-1711) and HF (High Five, *Trichoplusia ni*, ATCC: CRL-7701) cells were cultured in ESF (Expression Systems, CA, USA) medium supplemented with 2% FBS (fetal bovine serum) (ThermoFisher Scientific, Poland), 100 μg/ml streptomycin, 100 IU/ml penicillin, 10 μg/ml gentamycin, and 0.25 μg/ml amphotericin B. The culture was maintained in a humidified incubator at 27°C. Sf9 cells were used for baculovirus (BVs) generation and amplification, while HF cells were used for recombinant proteins expression.

LLC-Mk2 cells (*Macaca mulatta* kidney epithelial cells, ATCC: CCL-7) were maintained in minimal essential medium (MEM; two parts Hanks’ MEM and one part Earle’s MEM, ThermoFisher Scientific, Poland) supplemented with 3% FBS 100 μg/ml streptomycin, 100 IU/ml penicillin, and 5 μg/ml ciprofloxacin. The culture was maintained at 37°C under 5% CO_2_.

HAE (human airway epithelium) cultures were prepared and maintained as previously described [35]. Briefly, primary human tracheobronchial epithelial cells were obtained from airway specimens resected from patients undergoing surgery under Silesian Center for Heart Diseases. This study was approved by the Bioethical Committee of the Medical University of Silesia in Katowice, Poland (approval no: KNW/0022/KB1/17/10 dated on 16.02.2010). A written informed consent was obtained from all patients. Primary cells were expanded on plastic to generate passage 1 cells and plated on permeable Transwell inserts (6.5 mm-diameter) supports. HAE cultures were generated by provision of an air-liquid interface for 6-8 weeks to form well-differentiated, polarized cultures that resemble *in vivo* pseudostratified mucociliary epithelium.

### Constructs (plasmids and bacmids)

The codon-optimized genes encoding for M, E and S proteins were synthesized (Genart, ThermoFisher Scientific, Germany), delivered in pMA plasmids and subcloned to pFastBacDual plasmids (ThermoFisher Scientific, Poland). The nucleotide sequences of the *M*, *E* and *S* genes were deposited in the GenBank database under accession numbers MH050812, MH050811 and MH050813 respectively. HA peptide (YPYDVPDYA) encoding sequence was introduced to *M* and *E* genes by PCR primes (restriction sites underlined): M_fwd_: GAC GAA TTC ATG TCT AAC TCT TCC GTC CCC CTG, M_rev_: GAC AAG CTT TTA TTA GGC ATA GTC GGG GAC GTC GTA AGG ATA GAT CAG GTG CAG CAG CTT TTC C, E_fwd_: GCG GTA CCT TAG ACG TTC AGG ACT TCG GCG, E_rev_: GCC CCG GGA TGT ACC CGT ACG ACG TCC CTG ACT ACG CTT TCC TCA GGC TGA TCG ACG AC], whereas *GFP* was fused to the *S* gene by *Bsm*BI restriction and direct ligation in order to avoid PCR amplification of long templates (4 kbp for *S*). The obtained nucleotide sequences were verified by DNA sequencing. As monocistronic and bicistronic pFastBac plasmids were used, depending on experiments, the following denotation was adopted in the later text: (M + E) and (M-HA + E) for bicistronic vectors. Recombinant bacmids and baculoviruses were generated using BAC-TO-BAC system (ThermoFisher Scientific, Poland). Briefly, *Escherichia coli* DH10-Bac competent cells were transformed with recombinant pFastBacDual vectors and the isolated bacmid DNA was purified and transfected into Sf9 cells. After 6 days recombinant baculoviruses (rBVs) were harvested, then amplified and finally titrated using plaque assay method.

### SDS-PAGE and Western blot

Insect cells or culture media were harvested and resuspended in denaturing buffer containing 10% SDS and 5% β-mercaptoethanol, and boiled for 5 minutes (unless indicated otherwise). For detection of the S protein, samples were resolved by 8% Laemmli SDS-PAGE, for M protein by 12% Laemmli SDS-PAGE, for E protein by 16% Schagger/von Jagov SDS-PAGE. PageRuler and PageRuler Plus Prestained Protein Ladders (ThermoFisher Scientific, Poland) was used in this study as protein size marker. Gels were stained with Coomassie brilliant blue or subjected to electrotransfer in 25 mM Tris, 192 mM glycine, 20% methanol buffer onto an activated PVDF membrane. The membrane was blocked with 5% skim milk in Tris-buffered saline supplemented with 0.05% of Tween 20, followed by 1 hour incubation with rabbit polyclonal anti-M HCoV-NL63 serum (1:15,000, kindly provided by dr Lia van der Hoek) or mouse polyclonal anti-S HCoV-NL63 serum (1:40, Euogentec, Belgium) and respectively anti-rabbit (1:20,000, Dako, Denmark) and anti-mouse (1:20,000, Dako, Denmark) secondary antibodies conjugated with horse radish peroxidase (HRP). The signal was developed using Immobilon Western Chemiluminescent HRP Substrate (Millipore, Poland) and visualized by exposing the membrane to an X-ray film (ThermoFisher Scientific, Poland).

### Denaturation, deglycosylation and proteolysis assays

For assessment of M protein aggregation upon thermal denaturation, HF cells infected with (M+E) rBV were pelleted 72 hours post infection (p.i.), resuspended in 50 mM Tris, 100 mM NaCl buffer, with addition of 0.05% *N*,*N*-Dimethyldodecylamine *N*-oxide (LDAO). Aliquots of cell lysates or culture media were clarified by centrifugation, mixed with denaturing buffer containing 10% SDS and 5% β-mercaptoethanol and incubated for 10 minutes at room temperature, 50°C, or 95°C. In parallel aliquots of harvested culture medium were prepared identically. Next, samples were loaded onto SDS-PAGE gels and separated.

To verify M protein glycosylation, cell culture supernatant was collected 72 hours p.i. from HF culture infected with (M+E) rBV. An aliquot of culture supernatant was diluted with water and mixed with denaturation buffer (final concentration 0.5% SDS and 40 mM DTT). After a 10 minute incubation at room temperature, endoglycosidase H reaction buffer and endoglycosidase H (Promega, Poland) were added (final enzyme concentration 75 U/μl) and sample was incubated for 2 hours at 37°C. At the same time a control sample without endoglycosidase H was prepared.

Similarly, for VLP proteolysis experiment, cell culture supernatant was collected as described above. Trypsin was added to final concentration of 0.04% and samples were incubated at 37°C for 15 minutes in the presence or absence of SDS (final concentration of 0.1%). The reaction was stopped with complete protease inhibitor cocktail (Sigma-Aldrich, Poland).

Denaturation, deglycosylation and proteolysis samples were analyzed by Western blot with anti-M HCoV-NL63 polyclonal serum.

### Confocal microscopy

For assessment of protein co-localization in insect cells, HF cells were grown in 6-well culture plates on glass coverslips coated with 0.01% poly-L-ornithine in water (Sigma-Aldrich, Poland) for better cell adhesion. Cells were infected with rBVs at multiplicity of infection (MOI) of 1 and 48 hours p.i., fixed with 4% formaldehyde, permeabilized with 0.2% TritonX-100 in phosphate buffered saline (PBS), and blocked for 1 hour with 5% bovine serum albumin in PBS. Expression of M, M-HA, E, E-HA and S proteins was detected with rabbit polyclonal anti-membrane serum (1:1,000; 2 hours incubation), mouse anti-HA antibody (1:500, Antibodies Online, USA), and rabbit polyclonal anti-S serum (1:100, kindly provided by dr Lia van der Hoek), respectively. Anti-rabbit or anti-mouse antibodies conjugated with Alexa 488/546 at 1:400 dilution (Santa Cruz, USA) were used as secondary antibodies. Cell nuclei were stained with DAPI (0.1 g/ml in PBS; Sigma-Aldrich, Poland). Coverslips were mounted on glass slides with Prolong Diamond (Sigma-Aldrich, Poland) or Vectashield medium (Vector Laboratories, United Kingdom).

For transduction of VLPs into LLC-Mk2 cells or HAE cultures, LLC-Mk2 were grown to 80% confluence for 48 hours in 6-well culture plates on glass coverslips. HAE cultures were fully differentiated (as described above). Cells were then washed thrice with PBS and inoculated with 1 ml for LLC-Mk2 and 300 μl for HAE of VLPs-containing supernatants collected 72 hours p.i. from HF cultures. Next, LLC-Mk2 cells or HAE cultures were incubated for 3 hours at 32°C under 5% CO_2_ and further washed thrice with PBS. Subsequently, cells were fixed, permeabilized and stained as described above. Additionally, actin filaments were visualized with phalloidin conjugated with Atto 633 (0.132 μM, Sigma-Aldrich, Poland).

For ACE2 depletion, LLC-Mk2 cells were pre-incubated for 2 hours with phorbol 12-myristate 13-acetate (PMA, Sigma-Aldrich, Poland) diluted in culture medium to final concentration 10 μM, directly before VLP inoculation [36, 37]. ACE2 was detected with rabbit antibody (1:100, Abcam, UK), and secondary anti-rabbit antibody conjugated with Alexa 488 at 1:400 dilution (Santa Cruz, USA).

For HCoV-NL63 VLPs mediated antibody delivery, 500 μl aliquots of HA-tagged VLPs [(M-HA + E) +S] were incubated overnight, at 4°C with 5 μg of anti-HA mouse antibody. VLP-antibodies complexes were then added to LLC-Mk2 cells and left for 3 hours at 32°C under 5% CO_2_. After a triple wash with PBS slides were prepared as described above, with the exception of incubation with the primary antibody. Simultaneously, control cells were incubated with anti-HA antibody without HA-tagged VLPs.

Fluorescent images were acquired under a Leica TCS SP5 II confocal microscope (Leica Microsystems GmbH, Germany) with 60× 1.4 NA oil immersion objective and a Zeiss LSM 710 confocal microscope (Carl Zeiss Microscopy GmbH, Germany) with 40× 1.4 NA oil immersion objective. Images were acquired using Leica Application Suite Advanced Fluorescence LAS AF v. 2.2.1 (Leica Microsystems CMS GmbH, Germany) or ZEN 2012 SP1 software (Carl Zeiss Microscopy GmbH, Germany), respectively. All images were processed using ImageJ 1.47v (National Institutes of Health, Bethesda, Maryland, USA) with only linear adjustments of brightness and contrast. Z-stacks were deconvolved using AutoQuant X3 deconvolution software (Media Cybernetics).

### Electron microscopy

HF cells were co-infected with (M + E) rBV and S rBVs at MOI = 4 and cultured for 48 hours. For the ultrastructural analyses, cultured cells were fixed in Karnovski solution [2,5% glutaraldehyde / 2,5%formaldehyde (1:1) in PBS] at 4°C. After centrifugation at 800 rpm (3 minutes), cells were rinsed in 0.1 M phosphate buffer (pH 7.4) and fixed again in 1% buffered osmium tetroxide solution (Sigma-Aldrich, St. Louis, MO, USA) for 2 hours. In the next stage samples were rinsed in phosphate buffer and dehydrated in series of ethyl alcohol and acetone according to standard procedure. Dehydrated samples of culture were embedded in epoxy resin—Poly/Bed 812 Embedding Media/DMP-30 Kit (Polyscience, Inc., Warrington, PA, USA). The resin polymerization was conducted at 60°C for 72 hours. The ultrathin sections (80-nm thick) were contrasted with uranyl acetate (Polyscience, Inc., Warrington, PA, USA) and citrate lead (Sigma-Aldrich, St. Louis, MO, USA). The ultrastructural observations were performed by transmission electron microscope Hitachi H500 at an accelerating voltage of 75 kV.

For imaging of purified VLPs the suspension was fixed in Karnovski solution and inoculated onto a single-hole copper grids, the was grid coated with a support film (Formvar 15/95E, Sigma-Aldrich, St. Louis, MO, USA). After drying the material was stained with uranyl acetate (Polyscience, Inc., Warrington, PA, USA) and citrate lead (Sigma-Aldrich, St. Louis, MO, USA). Subsequently, the grids were washed with water and dried in air at room temperature. The ultrastructural observations were performed by transmission electron microscope Hitachi H500 at an accelerating voltage of 75 kV.

### Protein purification

HF cells were infected with (M+E) or (M-HA+E) and S rBVs at MOI = 4 and cultured for 72 hours. Untagged VLPs were harvested by centrifugation (5,000 × *g*, 30 min) of cell culture medium (secreted fraction) or by re-suspension of cells expressing tagged VLPs [(M-HA+E)+S] in the binding buffer (20 mM K_2_HP_4_O/KH_2_PO_4_ pH = 6.2, 70 mM NaCl) and disrupted by 3 rounds of mechanical homogenization (30 seconds, 8,000 rpm; Ultra Turrax, IKA, Germany) (intracellular fraction). Both fraction collected—secretory and intracellular—were clarified by centrifugation (5,000 × *g*, 30 min) and diluted 1:1 with the binding buffer. Samples were loaded onto 5 ml heparin HT column (GE Healthcare, Poland), connected to AKTA FPLC system (AKTA, Sweden). Before purification, the column was equilibrated with the binding buffer. Proteins were eluted with linear NaCl gradient (50 mM to 2 M NaCl in binding buffer) and the collected peak fractions were pooled and counter dialysed against 25 mM Tris pH 8, 50 mM NaCl, 1 mM EDTA buffer. The dialysate was subsequently loaded onto 1 ml CIM-QA monolith column (BiaSeparations, Slovenia) pre-equilibrated with dialysis buffer and connected to AKTA FPLC system. Again, proteins were eluted with a linear NaCl gradient (50 mM to 1 M NaCl in dialysis buffer). The collected peak fractions were analyzed using SDS-PAGE and Western blot with anti-M anti-S polyclonal sera.

### DLS

Chromatography purified fractions containing M protein were analyzed using dynamic light scattering (DLS). Hydrodynamic particle size measurements were done in ZEN2112 microcuvettes at 25°C using Zeta Sizer Nano S DLS instrument (Malvern Instruments, United Kingdom). Light scattering was measured 15 times at 10-second intervals for each sample. The data was analyzed using Zetasizer ver.7.11 software (Malvern Instruments, United Kingdom).

### *in cell* ELISA

HF cells were co-infected with (M-HA + E) rBV and S rBV at MOI of 4 and cultured for 72 hours. After this time, cells were mechanically disrupted in culture medium and cell debris was harvested by centrifugation (5,000 × *g*, 5 min). 1 ml portions of supernatants containing HA-tagged HCoV-NL63 VLPs were incubated overnight, at 4°C with 5 μg, 10 μg, or 20 μg of anti-HA mouse antibody, serially diluted and added to LLC-Mk2 cells seeded in 96-well culture plate 48 hours earlier. Cultures were incubated for 3 hours at 32°C under 5% CO_2_ and washed 4 times with PBS. Next, the cells were fixed with 4% formaldehyde, permeabilized with 0.2% Triton X-100 in PBS and blocked for 1 hour with 5% bovine serum albumin in PBS. The presence of anti-HA antibodies internalized together with VLPs was evaluated with secondary anti-mouse antibodies (1:5,000) conjugated with HRP. Signal was visualized with 100 μl of 3, 3', 5, 5'-tetramethylbenzidine (TMB, OptiEIA, BD, USA) substrate, and the reaction was stopped with 100 μl of 1 M HCl. Absorbance was measured at λ = 450 nm using Tecan Infinite 200 Pro microplate reader. All measurements were performed in triplicates and background (from control wells) was subtracted.

## Results

### Expression for HCoV-NL63 proteins in insect cells

**Fig 1:**
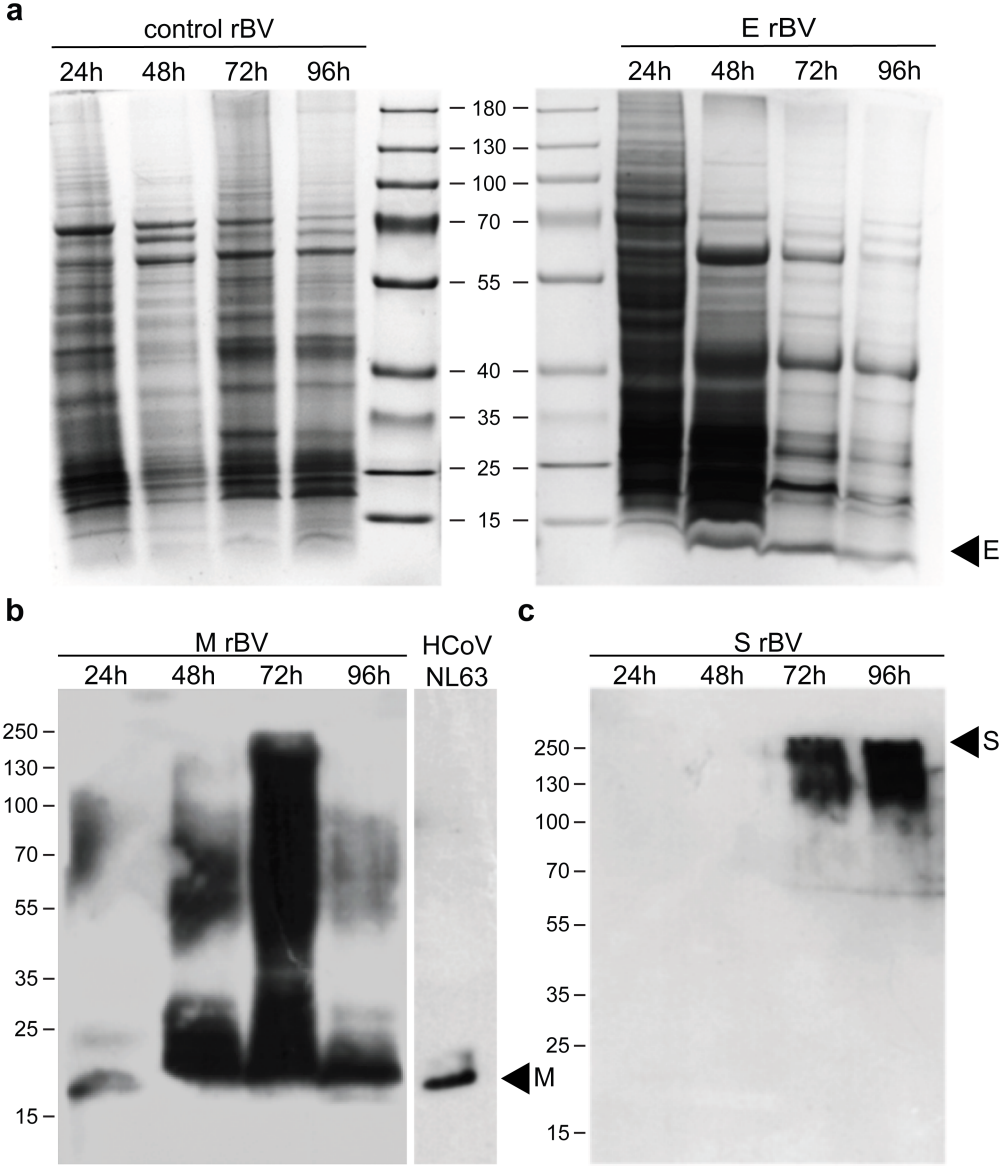
Expression of HCoV-NL63 proteins. HF cells were infected (MOI = 1) with E or M or S or control rBVs. Cells were harvested at different times post infection and resolved by 16% or 12% or 8% SDS-PAGE for the E, the M and the S proteins respectively. For E protein visualization gel was stained with Coomassie (**a**), whereas M and S protein were visualized by Western blot with anti-M (**b**) and anti-S antibodies (**c**), respectively. Inactivated HCoV-NL63 was used as positive control for the anti-M antibody.

HCoV-NL63 VLPs were produced using a baculovirus expression system as the most suitable for efficient expression of complex protein structures [38, 39]. For that, recombinant baculoviruses coding for M, E and S proteins of HCoV-NL63 were created. Additionally, M and E protein tagged with HA peptide and S protein fused with GFP were engineered. All sequences were codon-optimized for the expression in insect cells, synthesized and sub-cloned to one bicistronic or two monocistronic separate donor plasmids for more flexible regulation of protein expression [S1 Fig]. After isolation of rBVs, HF cells were infected at MOI 1 and cultures were harvested at different times post infection, to screen for the best expression conditions. Expression of the E protein was sufficient to be detected by Coomassie staining of the SDS-PAGE gel. Respective bands were visible in cell lysates collected 48, 72 and 96 hours p.i. [Fig 1a]. The M and the S proteins were more elusive, and their expression could only be confirmed by Western blot [Fig 1b and 1c].

**Fig 2:**
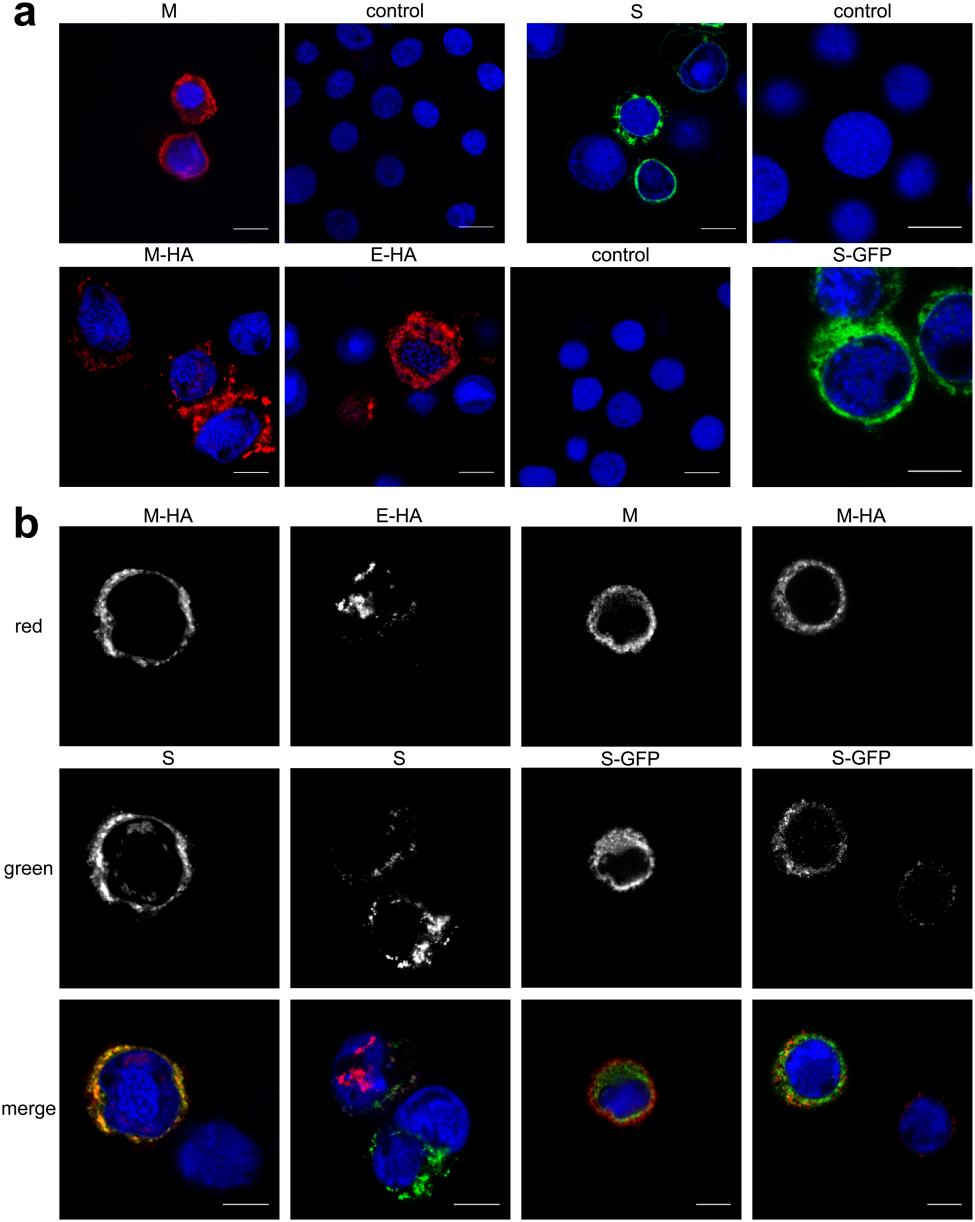
Expression and co-localization of HCoV-NL63 proteins in insect cells. Confocal microscopy analysis of HF cells infected with single rBVs: M, S, M-HA, E-HA, and S-GFP (**a**) or co-infected with several rBVs. Single channels images and merged pictures are shown (**b**). M was detected with anti-M antibody (shown in red); M-HA and E-HA proteins were detected with anti-HA antibody (shown in red); S was detected with anti-S antibody and S-GFP (shown in green); nuclei are presented in blue. Scale bar: 10 μm.

To further confirm expression of untagged and tagged M, E and S proteins in insect cells, samples were visualized with confocal microscopy. For this experiment, HF cells infected with M, M-HA, E-HA, S and S-GFP rBVs were grown on coverslips, fixed and stained with fluorophore conjugated antibodies. As shown in Fig 2a, protein expression was observed for all tested variants.

Next, we wanted to determine if tagged M, E and S proteins interact with each other within producer cells. For this, HF cells were co-infected with different monocistronic rBVs (coding for tagged or untagged proteins, as summarized in S1 Table) and examined for co-localization of HCoV-NL63 proteins. We observed that M-HA protein but not E-HA protein, co-localized with S protein, when co-expressed with E or M protein, respectively [Fig 2b]. This result suggests that HA tag on the E protein impairs its interaction with other proteins and thus precludes VLP formation. No co-localization could be detected for S-GFP with M or M-HA proteins (in presence of E protein), leading to conclusion that S-GFP is not incorporated into HCoV-NL63 VLPs.

In this step we have shown that expression of tagged and untagged M, E and S HCoV-NL63 proteins was successful. However, considering results of co-localization studies, tagged or untagged M protein and untagged variants of E and S rBVs were selected for further analysis.

### VLPs production and characterization

**Fig 3:**
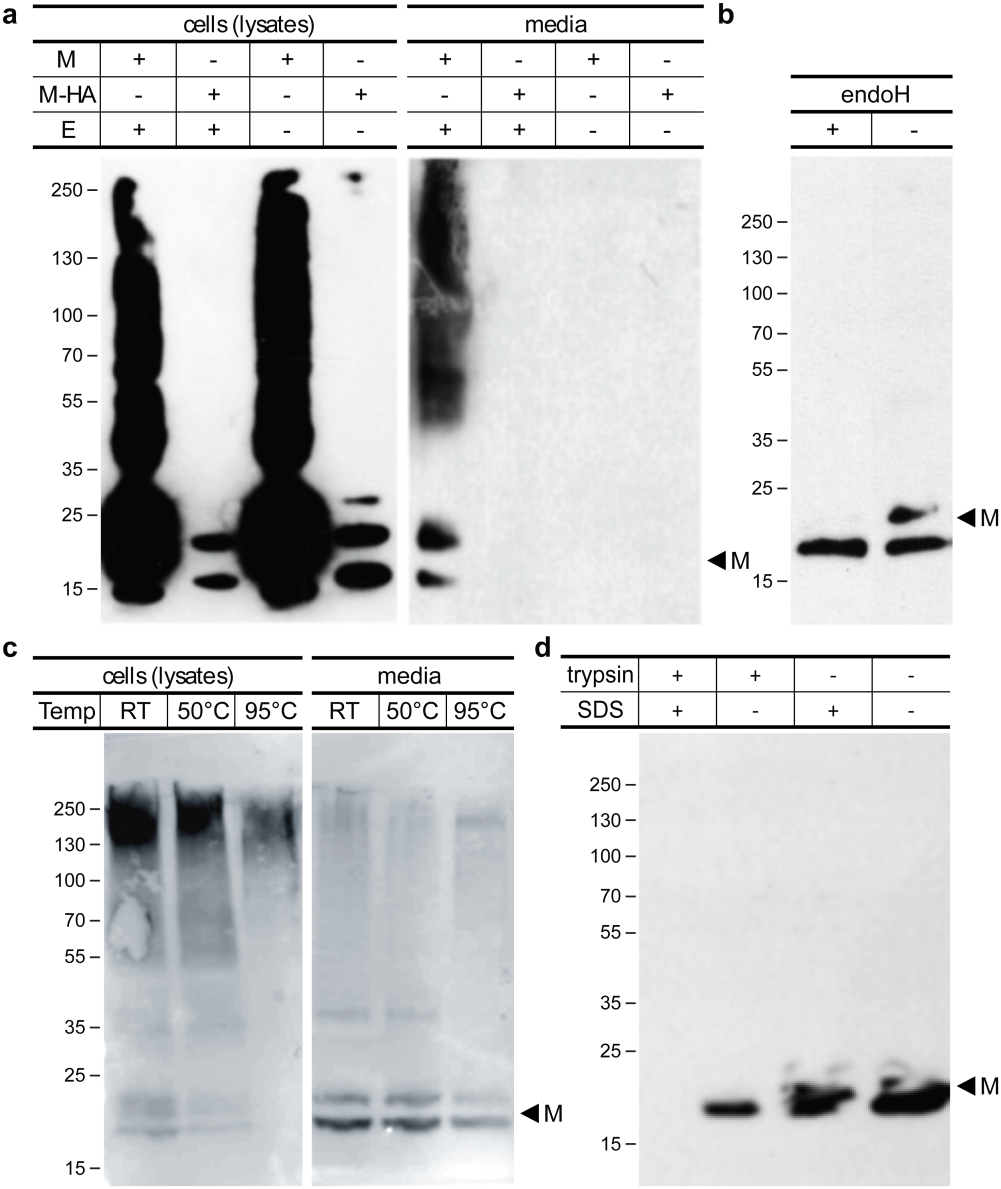
Characterization of HCoV-NL63 VLPs. Secretion of different variants of VLPs: HF cells were infected with (M + E), (M-HA + E), M and M-HA rBVs, cultured for 72 hours and harvested. Presence of M protein in respective cell lysates and culture media was assessed by Western blot (**a**). Deglycosylation assay: culture medium samples collected from cells infected with (M + E) rBV were incubated for 2 hours at 37°C with (+) or without (-) endoglycosidase H and analyzed by Western blot (with no thermal denaturation) (**b**). Thermal denaturation analysis: HF cells infected with (M + E) rBV were lysed, mixed with denaturing buffer containing 10% SDS and 5% β-mercaptoethanol, incubated for 10 minutes at room temperature (RT), 50°C or 95°C and analyzed by Western blot in parallel with harvested culture media (**c**). Protease resistance analysis: aliquots of supernatants containing (M + E) HCoV-NL63 VLPs were incubated with (+) or without (-) trypsin and SDS and analyzed by Western blot (**d**). In all experiments proteins were detected with anti-M antibody.

Having confirmed that HCoV-NL63 VLPs are properly expressed and co-localize in insect cells only when all three proteins are untagged, or when M protein is HA-tagged, we further investigated whether they were released to the culture medium. For this, media harvested from HF cells infected with bicistronic (M + E) and (M-HA + E) or monocistronic M and M-HA rBVs were analyzed by Western blot with anti-M antibody. This experiment revealed that M protein can be only detected in culture medium when it is co-expressed with the E protein. Additionally, HA tag not only decreases M protein expression levels but also impairs its release to the culture medium [Fig 3a]. However, functional VLPs were formed and could be isolated from the cells as shown in the downstream analyses. Co-infection of monocistronic M with E did not lead to detectable VLP secretion (data not shown). For this reason, bicistronic (M + E) and (M-HA + E) rBVs were used in subsequent experiments.

To further confirm that secreted M and E proteins were assembled into enveloped particles, their resistance to trypsin proteolysis was evaluated. As evidenced by Western blot, only in the presence of a detergent the M protein was fragmented by trypsin, demonstrating protection by the lipid envelope [Fig 3d].

Interestingly, we observed that standard thermal denaturation of samples resulted in M protein aggregation, as a high molecular mass product appeared when samples were heated prior to electrophoresis. This observation was true not only for cell lysates, but also for the secreted fraction of M protein, suggesting that the clogging effect does not depend on cellular localization. We have thus aimed to optimize the temperature and buffer content for preparation of the M protein for SDS-PAGE analysis [Fig 3c]. Although eliminating thermal aggregation, we could still detect several bands with anti-membrane antibody, most likely resulting from incomplete protein denaturation or the presence of differentially glycosylated M protein forms. To address this, we treated samples with glycosidases, what caused disappearance of the upper band, proving that at least a part of M protein was glycosylated [Fig 3b]. This result is consistent with observations described for other coronaviral M proteins [40–46].

**Fig 4:**
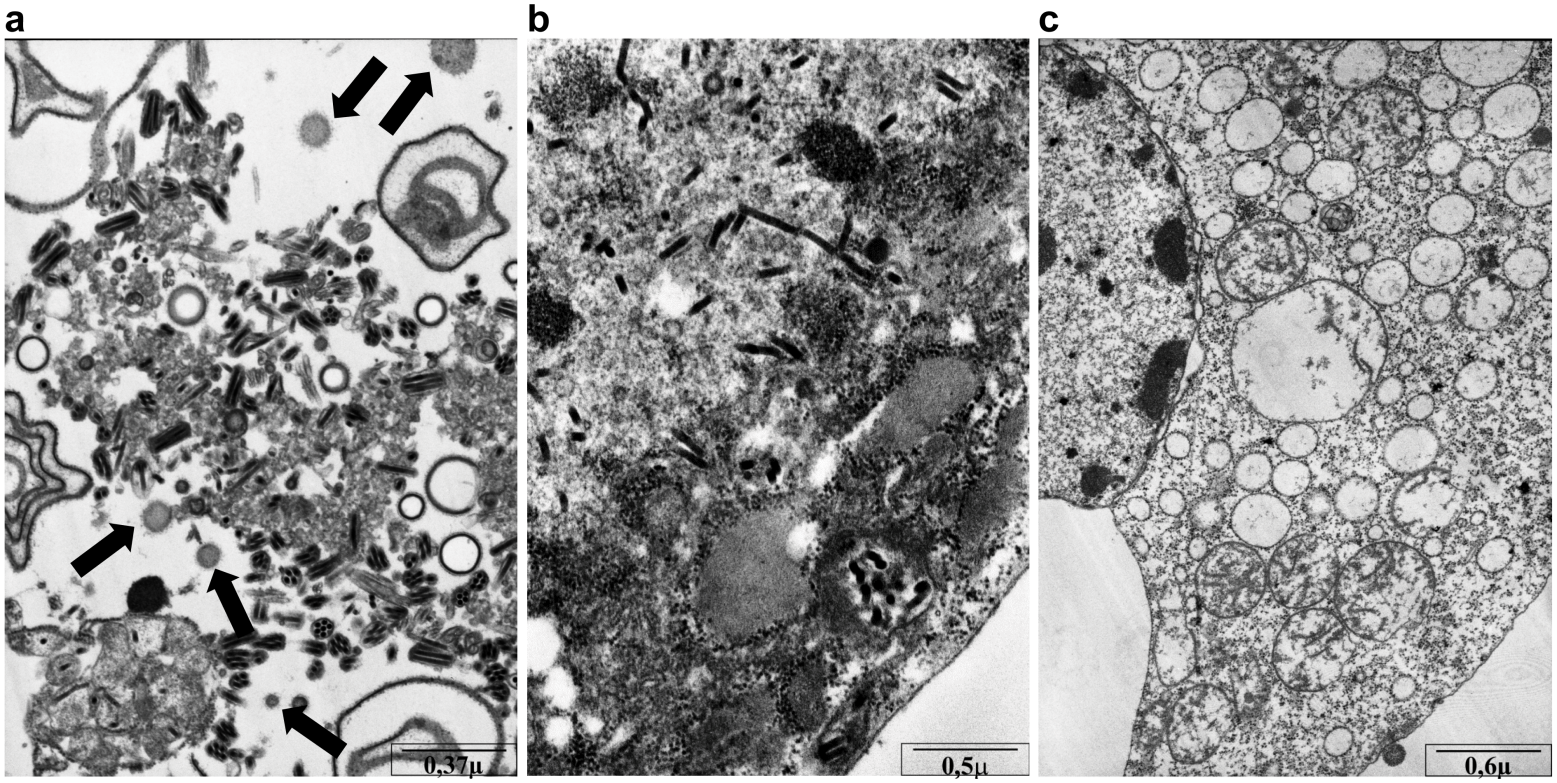
Transmission electron microscope images of HF cells expressing HCoV-NL63 VLPs. HF cells co-infected with (M + E) rBV and S rBV (**a**), infected with control rBV (**b**) and uninfected HF cells (**c**). Representative images are shown. Arrows indicate VLPs. Scale bars are presented in the lower right corner of each image.

Finally, to test whether the expressed proteins do assemble into VLPs, HF cells co-infected with (M + E) rBV and S rBV were examined under electron microscopy. Spherical enveloped particles, resembling the previously published coronaviral VLPs were observed in infected cells but not in control cells. Moreover, distinctive spike projection on the outer rim of these structures are visible. The diameter of putative VLPs ranged from 40 nm to 200 nm and these were found in the cytoplasm of cells undergoing apoptosis due to the baculovirus infection [Fig 4].

### VLP extraction and purification

**Fig 5:**
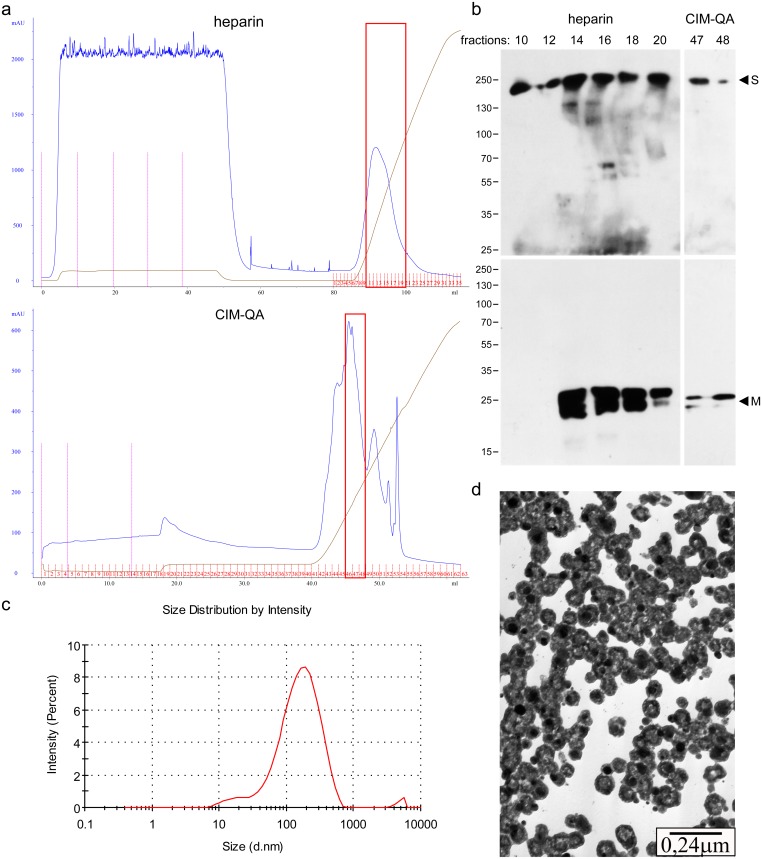
Purification of HCoV-NL63 (M + E) + S VLPs. Heparin (upper) and CIM-QA (lower) column purification profiles (**a**). Respective fractions were collected (indicated with frames) and analyzed with Western blot with anti-M and anti-S antibodies (**b**). DLS analysis of purified HCoV-NL63 VLP fraction (**c**). Electron micrograph of purified VLPs: collected fractions were fixed, immobilized on copper grids and positively stained, as described in the Materials and Methods section (**d**).

Culture media from insect cells infected with (M + E) and S rBVs or cell homogenates obtained from cells infected with (M-HA + E) and S rBVs were harvested and purified on heparin column, as described in Materials and Methods. Heparin column purification allowed to remove majority of contaminants, including albumin from HF culture medium, but VLPs co-purified with other proteins, most likely originating from BVs. Samples were thus submitted for the second stage of purification using CIM-QA monolith column recently reported to be a useful tool for virus and VLPs preparations [47–49]. This step allowed for further fractionation of the material collected from heparin column. Subsequent Western blot analysis showed the presence of M and S proteins in the same fractions [Fig 5a and 5b]. Homogeneity of the obtained material was assessed by Dynamic Light Scattering. Hydrodynamic particle size measurements indicate that the mean diameter of obtained VLPs is 180 nm with a mean polydisperisty index (PdI) of 0.5 [Fig 5c]. Transmission electron microscopy analysis further confirmed that collected fractions contained intact VLPs [Fig 5d].

### VLP internalization into target cells

**Fig 6:**
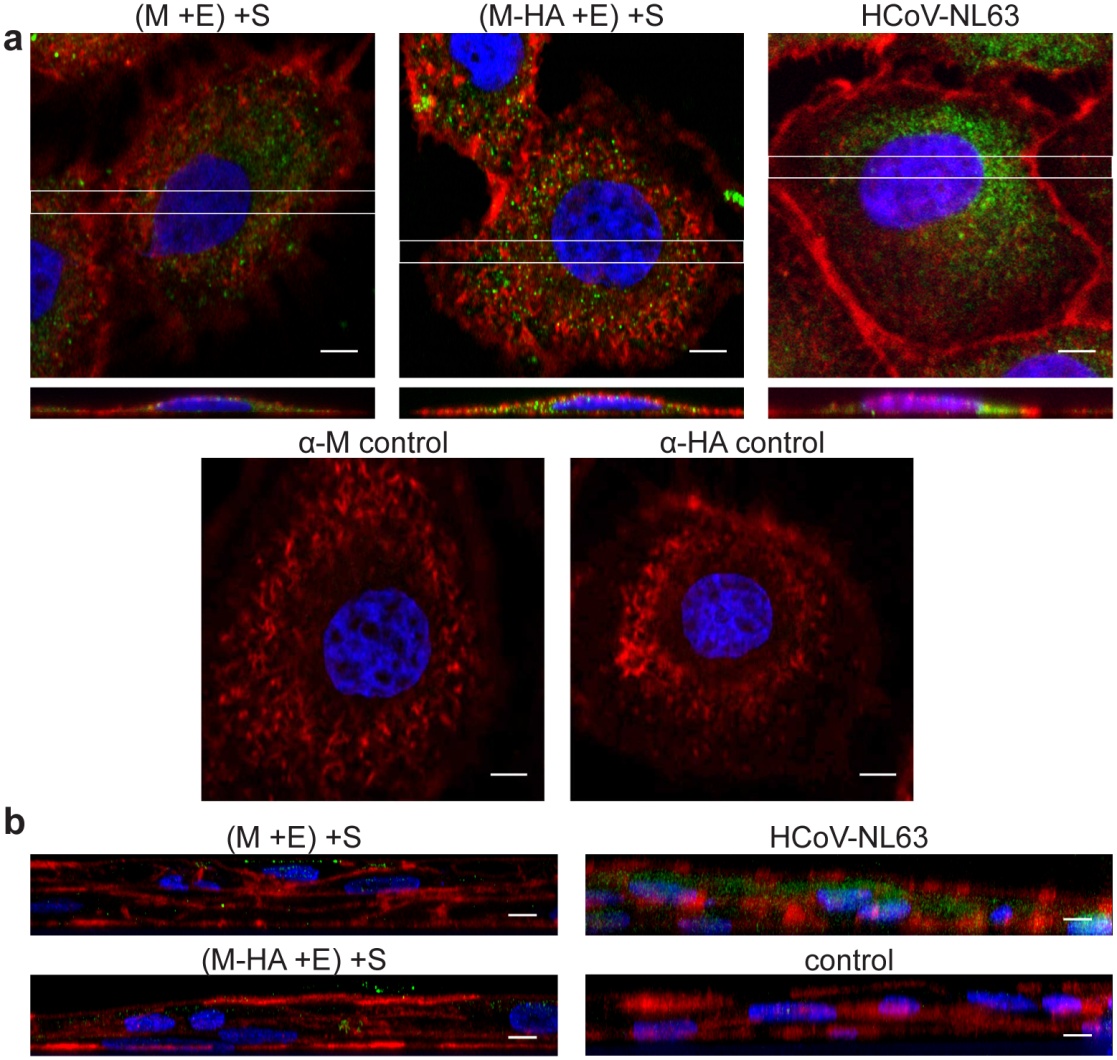
HCoV-NL63 VLPs enter susceptible cells. LLC-Mk2 (**a**) cells and HAE cultures (**b**) were incubated with (M + E) + S VLPs, (M-HA + E) + S VLPs, HCoV-NL63 and mock. Cells were then immunostained and analyzed by confocal microscopy (Z-stacks). Rectangles indicate areas of xz plane seen in orthogonal views (**a**). Images of HAE are orthogonal views created by maximum projection of axial planes (**b**). Actin fibers are shown in red, nuclei are shown in blue, VLPs and HCoV-NL63 are visualized in green. (M-HA + E) + S VLPs in LLC-Mk2 cells were detected with anti-HA antibody, whereas in other preparations VLPs and HCoV-NL63 were detected with anti-M antibody. Scale bar: 5 μm.

**Fig 7:**
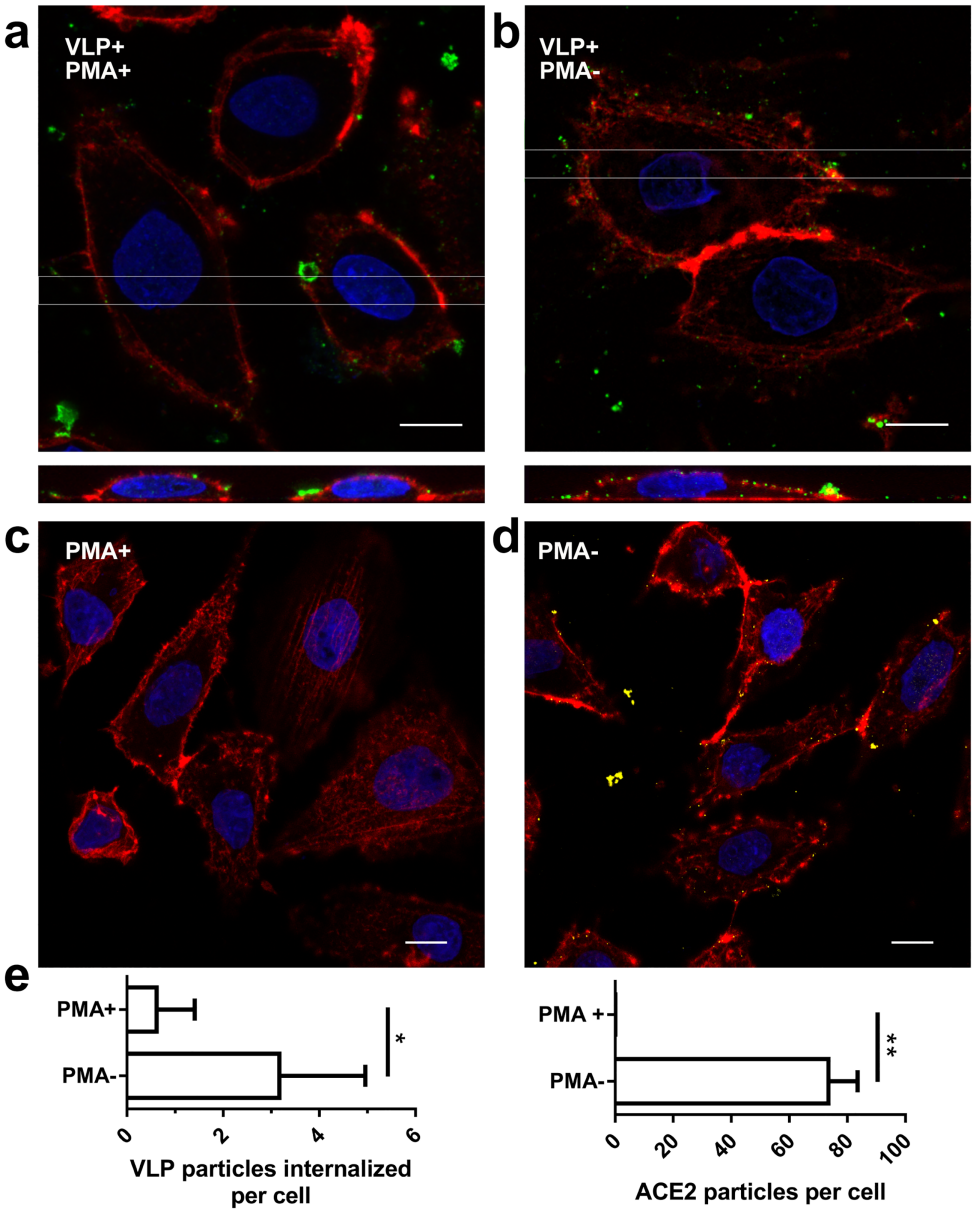
PMA treatment affects entry of HCoV-NL63 VLPs entry to the cell. LLC-Mk2 cells were transduced with (M + E) + S VLPs with (**a**) or without (**b**) PMA pre-treatment, immunostained and analyzed by confocal microscopy (Z-stacks). Rectangles indicate areas of xz plane seen in orthogonal views. ACE2 molecule was additionally stained in PMA-treated (**c**) or untreated LLC-Mk2 cells (**d**). Actin fibers are shown in red, nuclei are shown in blue, M protein is detected with anti-M antibody and visualized in green whereas ACE2 receptor is detected with anti-ACE2 antibody and visualized in yellow. Each image is a single confocal plane (xy) with its orthogonal view (xz) created by maximum projection of axial planes (thickness 3 μm). Scale bar: 10 μm. Number of VLPs internalized to LLC-Mk2 cells and the ACE2 relative quantity per cell after PMA treatment or mock-treatment was determined and is presented in graph: * *P* < 0.05; ** *P* < 0.01 (**e**).

We investigated the capacity of HCoV-NL63 VLPs to transduce target cells. For this, LLC-Mk2 cells, permissive for HCoV-NL63 infection, were incubated with the VLPs: (M + E) + S or (M-HA + E) + S. Fixed samples were submitted for confocal imaging. Reconstruction of the orthogonal view confirmed that tagged and untagged HCoV-NL63 VLPs were effectively internalized into LLC-Mk2 cells [Fig 6a]. Same procedure was carried out for HAE cultures where HCoV-NL63 VLPs internalization was detectable, but less efficient than in LLC-Mk2 cells [Fig 6b], what is consistent with our previous reports on the virus [50]. Obtained results were promising, but to assess the specificity of the process we tested whether VLPs are also internalized into cells deprived of the natural receptor—ACE2, as we have previously shown, that this is not the case for the virus [50]. For this, LLC-Mk2 cells were pre-treated with PMA, known to deplete ACE2 from the cell surface [36, 37, 50] and incubated with the obtained VLPs and analyzed by confocal microscopy. VLPs adhesion, but not penetration into target cells pre-treated with PMA was observed, proving that the obtained VLPs specifically penetrate cells expressing the entry receptor [Fig 7].

### VLP—Mediated protein delivery into target cells

Knowing that VLPs can transduce target cells, we asked the question whether they can be used to deliver a cargo. To address this, we used HA-tagged VLPs decorated with anti-HA tag antibodies for two reasons: contrary to anti-M and anti-S polyclonal sera the anti-HA antibody is monoclonal and thus specificity of the VLP—antibody formation is higher and secondly, attaching a heterologous moiety to M or S protein could obstruct receptor interacting domains and thus inhibit VLPs’ cell penetration.

**Fig 8:**
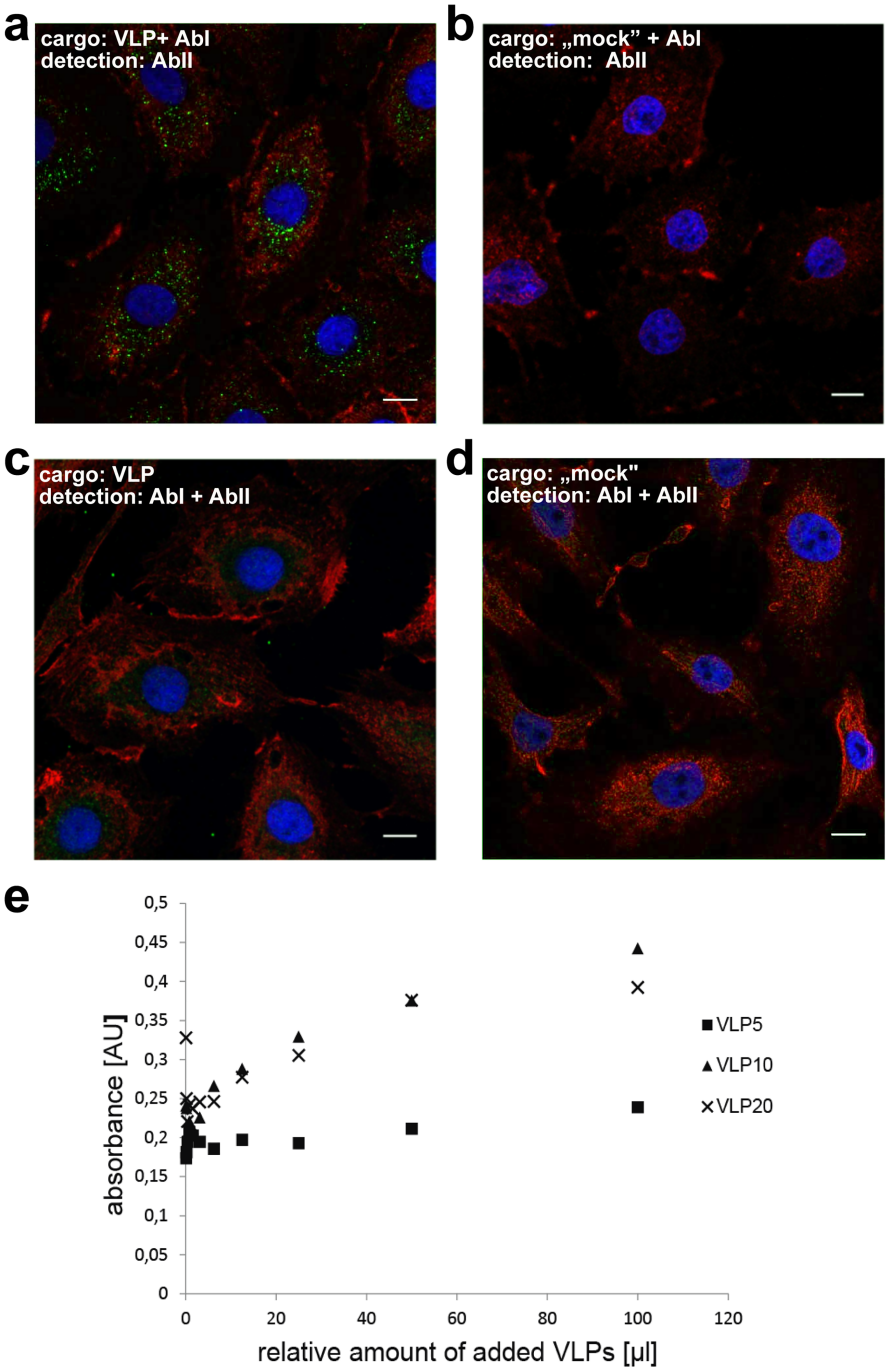
HCoV-NL63 VLPs mediated delivery to target cells. Confocal microscopy analysis of LLC-Mk2 cells transduced with HA-tagged VLPs pre-incubated with anti-HA antibody (**a**), anti-HA antibody alone (**b**) HA-tagged VLPs alone (**c**) or control (mock-incubated) cells (**d**). AbI denotes primary antibody (anti-HA tag) and AbII denotes secondary antibody, conjugated with the fluorophore. Actin fibers are shown in red, nuclei are shown in blue, and HA tag is visualized in green. Scale bar: 10 μm. ELISA detection of anti-HA antibody transported to LLC-Mk2 cells by HA-tagged HCoV-NL63 VLPs. VLP5, VLP10 and VLP20 denotes the amount of anti-HA antibody (5, 10, and 20 μg, respectively), used during pre-incubation with HA-tagged HCoV-NL63 VLPs (**e**).

To ensure complex formation (M-HA + E) + S VLPs were pre-incubated with different amounts of anti-HA antibody and then overlaid on LLC-Mk2 cells. Simultaneously, control cells were incubated with the same amount of the anti-HA antibody, to rule out the possibility of VLP-independent internalization. The cargo internalization was evaluated with a fluorescently labeled secondary antibody and visualized with confocal microscopy [Fig 8a–8d]. A signal was clearly detected only in preparations were VLPs were present, making evidence that HCoV-NL63 VLPs not only efficiently transduce model target cells, but may also serve as carriers for relatively large proteins.

To further analyze if antibody delivery to target cells is dose-dependent, we performed an *in cell* ELISA. HA-tagged VLPs were pre-incubated with various amounts of anti-HA antibody, serially diluted and overlaid on LLC-Mk2 cells in a microtiter plate. The relative amount of the transduced anti-HA antibody was assessed using the standard ELISA protocol. As depicted in Fig 8e, the measured absorbance was proportional to the concentration of VLP-antibody complexes. Interestingly, the detected signal was similar for doses of 10 μg and 20 μg of antibody pre-incubated with VLPs suggesting that the interaction of the ant-HA antibody and the HA tag on the VLP surface has been saturated.

This result is a proof of concept for the development of the HCoV-NL63 VLP as a protein delivery platform.

## Discussion

In this manuscript we describe the production of HCoV-NL63 VLPs by expression of the viral structural proteins in insect cells. By co-expression of the M, E and S proteins we have obtained functional particles displaying a typical corona-like structure. Minimal protein requirement for coronaviral particle assembly is controversial (summarized in S2 Table). Some groups postulate that M and E proteins are sufficient for formation of VLPs [4, 5, 9], whereas others report the critical role of the N protein in this process [8, 51]. Obtained results suggests that unlike most other enveloped RNA viruses, coronaviruses employ a nucleocapsid-independent strategy to drive assembly and budding, but the stabilizing role of this protein cannot be excluded [6, 9, 12]. The combination of proteins essential for VLPs formation seem to also depend on the viral species and the expression system used.

Expression of the HCoV-NL63 soluble proteins in insect cells was successful, yet we observed that standard treatment of the sample during preparation for SDS-PAGE resulted in M protein aggregation and hinder detection of the protein. This finding is in accordance with Lee *et al*. describing thermal aggregation of SARS-CoV membrane protein [52]. Interestingly thermal aggregation of M protein was not reported for MHV, TEGV, IBV, and other publications concerning SARS-CoV.

HCoV-NL63 VLPs assemble in insect cells and are released to the culture medium upon M and E protein expression. S protein was incorporated to VLPs when present, but we did not observe its effect on VLP expression level or stability, what is consistent with reports concerning other coronaviruses (reviewed in [53]). Further, we also investigated the formation and secretion of tagged HCoV-NL63 VLPs. Interestingly, we found that HA peptide tagged M protein was still assembly competent and the (M-HA + E) + S VLPs were functional but were not released from producer cells. It should be pointed out that HA peptide was fused to the C terminus of the M protein, which is considered to be the essential for M-M interaction and VLP assembly [10, 12, 18, 29, 54, 55]. Several groups reported that deletion, substitution or extension of the C terminus of the M protein affects interaction with other proteins [6, 8, 18, 56–58]. M protein of most coronaviral species exposes its N-terminus outside the virion, whereas the C-terminal tail is hidden inside the particle (N_exo_-C_endo_ orientation). Only TEGV M protein was shown to adopt two topologies: N_exo_-C_endo_ and N_exo_-C_exo_ orientation [59]. Accordingly, assembly of TEGV VLP containing M protein with HA tag fused to the C terminus has been reported [16]. Topology of the HCoV-NL63 M protein has not been assessed yet, but one may assume that alphacoronaviral M proteins may adopt N_exo_-C_exo_ topology allowing for C-terminal modification. Here we show successful VLP-mediated delivery of the tag-specific antibody directed to the HA-tag fused to the C terminus of the M protein, what supports this assumption.

VLPs are routinely purified by density gradient ultracentrifugation, which is a tedious and long procedure. Our efforts to purify HCoV-NL63 VLPs using this method failed. Most of the alternative purification methods are based on size-exclusion column purification [60–62]. A limitation of this approach is that a relatively low amount of material can be loaded onto a size-exclusion column. Based on our previous finding that heparan sulfate serves as an attachment receptor for HCoV-NL63 [50], we reasoned that HCoV-NL63 VLPs may bind to a heparin column. Moreover, heparin can act as a weak cation exchanger and it was shown to interact with a number of viruses [63–65]. Employment of the heparin column was successful and allowed for partial purification of HCoV-NL63 VLPs. The quality of preparation was high, but unfortunately BVs co-purified with VLPs (data not shown). This observation is in agreement with known BVs affinity for heparin columns [66]. However, this problem was solved by further fractionation using monolith ion exchange column. Even though the yield of the whole procedure could not be calculated for the analytical scale preparation, this two-step chromatography purification method was validated by verifying transducing capacities of the purified VLPs and thus confirming their integrity. Considering the obtained results, the described protocol seems to be an attractive option for VLPs that are burdensome for ultracentrifugation.

Last but not least, one of the main reasons for development of HCoV-NL63 VLPs was to create a vector that exhibits a narrow tissue tropism. HCoV-NL63 VLPs is a good candidate, as the parental virus infects only ciliated cells of the human airway epithelium expressing the ACE2 protein. Even though numerous works describe successful production of SARS based VLPs potentially displaying the same tropism, only one study shows that SARS VLP cell entry is mediated by ACE2 [67]. Here, we demonstrated that HCoV-NL63 VLPs not only penetrate human airway epithelium, but also that the presence of the ACE2 receptor on cell surface is a pre-requisite for the internalization. Further, we provided evidence that HCoV-NL63 VLPs can serve as a carrier for heterologous proteins such as antibodies. We have shown that developed VLPs may serve as a carrier for antibodies, similarly as previously described by Fender and colleagues for adenovirus-like particle mediated antibody delivery to HeLa cells [68]. We also attempted to anchor the cargo protein inside the VLP, by fusion of the GFP tag to the C-terminus of the S protein. The feasibility of such vector modification was described for MHV [69]. Unfortunately, HCoV-NL63 S-GFP proteins were not incorporated into VLPs, as no co-localization with M protein was observed. It is conceivable that a C-terminally fused tag may hinder M-S interactions or alter S subcellular localization and further studies are required.

Summarizing, we have successfully designed, produced and characterized HCoV-NL63 VLPs. Developed particles require the ACE2 protein for successful entry to the cell, thus may be used for delivery of nucleic acids, proteins or small molecules to the ciliated cells lining the conductive airways. Noteworthy, these particles could be potentially inhaled, thus overcoming the immune reaction related to systemic administration.

## Supporting information

S1 Fig Baculovirus constructs. Polh (polyhedrin) and p10—promoters in baculovirus sequence for protein expression in insect cells. M—membrane, E—envelope, S—spike. Dark grey rectangle represents GFP tag and light grey rectangle represent HA tag.
(TIF)

S1 Table Recombinant baculoviruses (monocistronic) used for co-infection of HF cells in co-localisation analysis. (DOCX)

S2 Table Minimal protein requirements for CoV VLP formation. (DOCX)
