# Supplementary figures and images for: Novel coronavirus-like particles targeting cells lining the respiratory tract
Source: PLoS One. 2018 Sep 5;13(9):e0203489. doi: 10.1371/journal.pone.0203489 (PMC6124810; doi:10.1371/journal.pone.0203489)

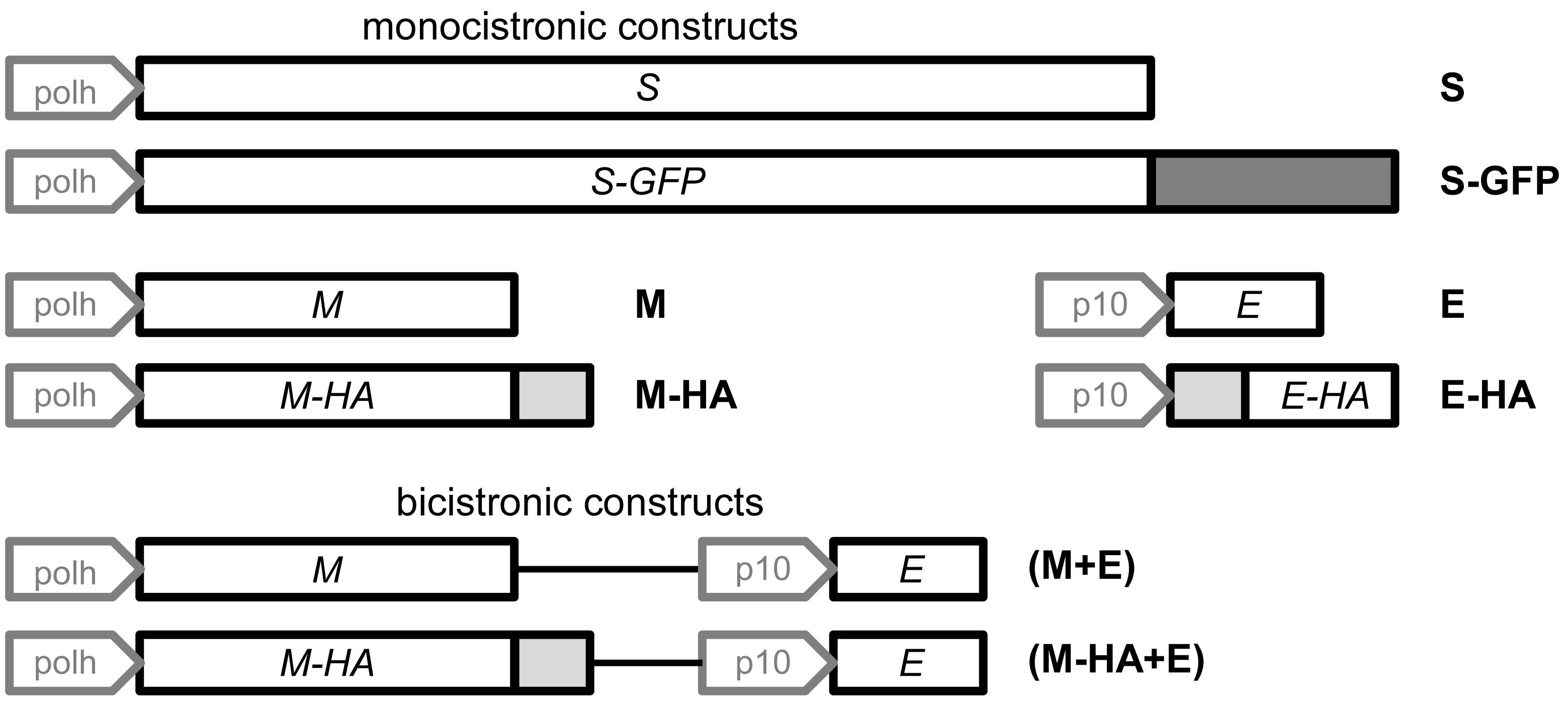

Supplement: S1 Fig — Polh (polyhedrin) and p10—promoters in baculovirus sequence for protein expression in insect cells. M—membrane, E—envelope, S—spike. Dark grey rectangle represents GFP tag and light grey rectangle represent HA tag. (TIF) [file pone.0203489.s001.tif]
