# Supplementary material for: Novel coronavirus-like particles targeting cells lining the respiratory tract
Source: PLoS One. 2018 Sep 5;13(9):e0203489. doi: 10.1371/journal.pone.0203489 (PMC6124810; doi:10.1371/journal.pone.0203489)
Supplement: S1 Table — (DOCX) [file pone.0203489.s002.docx]

**S1 Table. Recombinant baculoviruses (monocistronic) used for co-infection of HF cells in co-localisation analysis.**

| **rBVs used for co-infection** | **immunostaining** | **co-localisation** |
| --- | --- | --- |
| M-HA + E + S | M-HA and S | yes |
| M + E-HA + S | E-HA and S | no |
| M + E + S-GFP | M and S-GFP | no |
| M-HA + E + S-GFP | M-HA and S-GFP | no |
