# Supplementary material for: Novel coronavirus-like particles targeting cells lining the respiratory tract
Source: PLoS One. 2018 Sep 5;13(9):e0203489. doi: 10.1371/journal.pone.0203489 (PMC6124810; doi:10.1371/journal.pone.0203489)
Supplement: S2 Table — (DOCX) [file pone.0203489.s003.docx]

**S2 Table. Minimal protein requirements for CoV VLP formation.**

| **genus** | **CoV** | **expression system** | **proteins required for VLPs formation** | **reference** |
| --- | --- | --- | --- | --- |
| α | TGEV | mammalian cells, vaccinia virus | M, E | (Baudoux, et al., 1998) |
| β | SARS | insect cells, baculovirus | M, E | (Mortola and Roy, 2004) |
|  |  |  | M, E | (Ho, et al., 2004) |
|  |  | mammalian cells, plasmid based | M, N | (Huang, et al., 2004) |
|  |  |  | M, E | (Hsieh, et al., 2005) |
|  |  |  | M, E, N | (Siu, et al., 2008) |
|  |  |  | M, N | (Nakauchi, et al., 2008) |
|  |  |  | M | (Tseng, et al., 2010) |
|  | MHV | mammalian cells, vaccinia virus | M, E | (Vennema, et al., 1996) |
|  |  |  | M, E | (de Haan, et al., 1998) |
|  |  |  | M, N | (Narayanan, et al., 2000) |
|  |  |  | M, E | (Arndt, et al., 2010) |
|  |  | mammalian cells, plasmid based | M, E, N | (Boscarino, et al., 2008) |
| γ | IBV | mammalian cells, vaccinia virus | M, E | (Corse and Machamer, 2000) |
